# Supplementary material for: Blik is an extensible 3D visualisation tool for the annotation and analysis of cryo-electron tomography data
Source: PLoS Biol. 2024 Apr 30;22(4):e3002447. doi: 10.1371/journal.pbio.3002447 (PMC11268629; doi:10.1371/journal.pbio.3002447)
Supplement: S1 Tutorial blik tutorial — (PDF) [file pbio.3002447.s001.pdf]

# `blik` tutorial

Moritz A. Kirchner[1] and Lorenzo Gaifas[1]

[1]Institut de Biologie Structurale, Université Grenoble Alpes, CEA, CNRS, IBS, Grenoble, France.

`blik` is a plugin for the scientific visualisation software `napari`, bringing extra functionality for cryo-electron tomography (cryo-ET) data visualisation, manipulation and analysis. In this tutorial, you will learn the basics of `napari` and how to use the various tools provided by `blik`. When available, links to more extensive external documentation are provided throughout this tutorial.

## Contents

**1 `napari` basics** **2**

**2 Getting started with `blik`** **3**

**3 Opening and visualising data** **3**
3.1 Saving or writing data to a file . . . . . . . . . . . . . . . . . . . . . . . . . . 4
3.2 Visualising tomograms . . . . . . . . . . . . . . . . . . . . . . . . . . . . . 4
3.3 Visualising particles . . . . . . . . . . . . . . . . . . . . . . . . . . . . . . . 7

**4 Using `blik` to segment and pick** **7**
4.1 Tomogram segmentation . . . . . . . . . . . . . . . . . . . . . . . . . . . . 7
4.2 Manual picking . . . . . . . . . . . . . . . . . . . . . . . . . . . . . . . . . 8
4.3 Surface picking . . . . . . . . . . . . . . . . . . . . . . . . . . . . . . . . . 8
4.4 Filament picking . . . . . . . . . . . . . . . . . . . . . . . . . . . . . . . . 10
4.5 Resampling features . . . . . . . . . . . . . . . . . . . . . . . . . . . . . . 11

**5 Inspecting particle metadata** **12**

**6 Creating custom widgets** **13**

# 1 napari basics

napari is a multi-dimensional image viewer, focused on scientific image analysis. It provides both a graphical and programmatic interface to offer intuitive interaction and powerful customisation capabilities. napari is easily installed and can be extended with a growing number of plugins dedicated to specific image analysis tasks. You can find the full napari documentation and a usage guide at the napari tutorial page.

A variety of image formats can be loaded into napari and additional format support can be achieved by plugins (such as blik for cryo-ET related formats). Images can be opened from the command line, by dragging them into the napari window, or by selecting them from the *File > Open File(s)* (Ctrl+o) menu. The image will be loaded as a new Layer, the basic object that can be visualised in the napari viewer (similarly to layers in image-editing software like Photoshop ad GIMP). There are several layer types (e.g. Image, Surface, etc.), which all come with their specific layer controls, displayed at the top left of the viewer when a layer is selected (Figure 1).

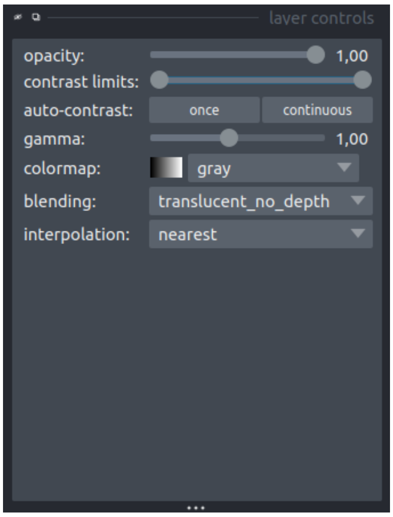


***Figure 1:*** *Layer controls panel for the* Image *layer, providing several basic controls such as contrast limits, colormapping and interpolation.*

Using this window, we may adjust basic properties like image contrast and colormap, surface colors, and others. If multiple layers are present, the layer list just below the layer controls can be used to reorder layers and select a specific layer to control. Layers can also be shown and hidden from this panel by clicking on the eye icon (Figure 2).

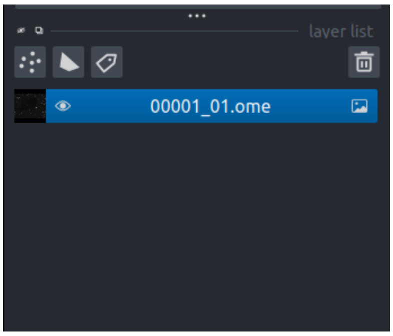


***Figure 2:*** *Layer list widget containing a single* Image *layer. On the left, the eye icon used to toggle layer visibility.*

In addition to the graphical controls, napari provides a built-in IPython console. To open the console, click the >_ symbol in the button row at the bottom left of the viewer (Figure 3).

The console can be used for on-the-fly programmatic interaction with the data via the `napari` python API.

The button row below the layer list also allows to toggle between 2D and 3D, as well other viewer controls.

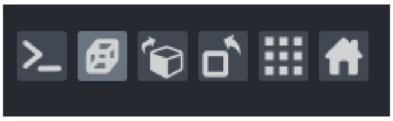

***Figure 3:*** *Bottom button row. In order: toggle ipython console, toggle 2D/3D, transpose dimensions, rotate visualisation, grid mode, reset view.*

`napari` is designed to handle n-dimensional data. When loading data with more than two dimensions, the `dimension slider` will open beneath the canvas. This slider can be used to scroll through the dimensions currently not displayed. The number of sliders depends on the dimensionality of the data: one slider for each dimension beyond the ones displayed. The play button on the left side of each slider allows to automatically scroll through that dimension.

More in-depth information on how to navigate the `napari` user interface (UI) can be found on the viewer documentation.

# 2 Getting started with `blik`

`blik` is a `napari` plugin for reading, writing, and interacting with cryo-ET and subtomogram averaging (STA) data (Gaifas et al. – *bioRxiv* 2023). This tutorial provides some basics for visualisation and annotation of cryo-ET data using `blik`; the full documentation can be found at https://brisvag.github.io/blik/.

There are multiple ways in which `blik` can be installed. If `napari` is already installed, you can install blik through the `napari` plugin manager (in `napari` UI go to *Plugins > Install/Uninstall Plugins...* and then search for `blik`), directly through pip. Alternatively, you can install `blik` and `napari` together using:

```
pip install "blik[all]"
```

This will also install additional plugins which are needed or segmentation interpolation and properties inspection.

The nightly build is updated more frequently with new features and uses the latest, unreleased `napari` (with some instability to be expected). To install the latest nightly build, follow the instructions on the readme.

# 3 Opening and visualising data

`blik` takes care of the data loading once installed; to open files, you can use the standard `napari` methods described above.

For images, `blik` supports many standard file formats such as `.mrc` (including `.mrcs`, `.st` and `.map`) but also formats like Dynamo `.em`, among others. This allows visualisation not only of tomograms, but also raw movies, tilt series and even things like output power spectra from ctffind4.

For particle data, `blik` takes Relion `.star`, Dynamo `.tbl` and a variety of other input formats format.

blik also comes with a file reader implemented as a napari UI widget (*Plugins > blik > file reader*), which offers more granular control over the loading process.

Once opened, the data will initially be loaded into a hidden layer: experiments can be selected and rendered visible through the main widget, or layers can be manually marked as visible from the layer list.

## 3.1 Saving or writing data to a file

To save any napari layer using a plugin, we simply need to select the layer of interest and hit *Ctrl + s* or go to *File > Save selected Layer(s)*. Depending on the layer type, napari will provide various output formats, among which those implemented by blik (such as .mrc for images, or .star for particles).

## 3.2 Visualising tomograms

Let's see what we can do with our data after loading it into blik. For this example, we are using a tomogram of *E. coli* minicells showcasing a sizeable chemosensory array underneath their membrane (Figure 4, Burt et al. – *Nature Communications* 2020).

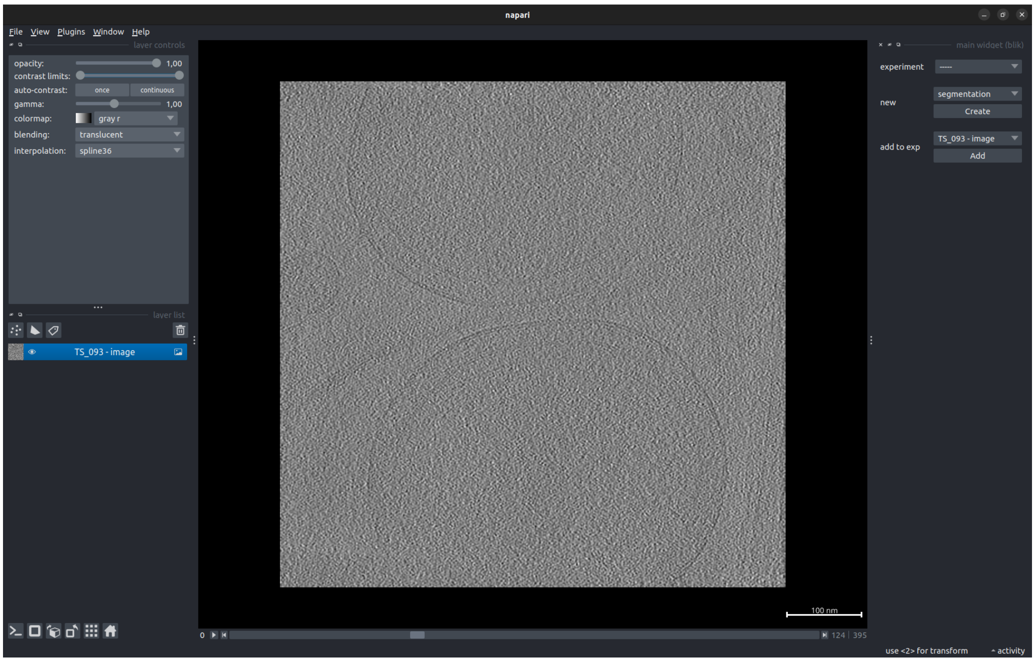


***Figure 4:*** *Z-slice through a (noisy) tomogram of* E. coli *minicells.*

At first glance, the low contrast of this tomogram makes it hard to segment or pick particles. To improve interpretability of the data, blik provides a gaussian filter, which can be applied to 2D images and slices with adjustable kernel size and sigma (Figure 5). The filter can be applied through its widget, accessible from the main menu.

If using the nightly build, to further improve contrast, we can increase the thickness over which the currently viewed slice is averaged using the slider on the main widget.

If we switch to the 3D view, the data will initially be depicted as a 2D slicing plane through the 3D volume. We can also adjust the thickness of this slice using the slider in the layer controls. We can also hold the shift key while dragging the plane up and down to slice through the tomogram volume.

We can change the alignment of the slicing plane through the volume to x or y by clicking on the corresponding direction under plane normal in the layer controls. Using multiple layers

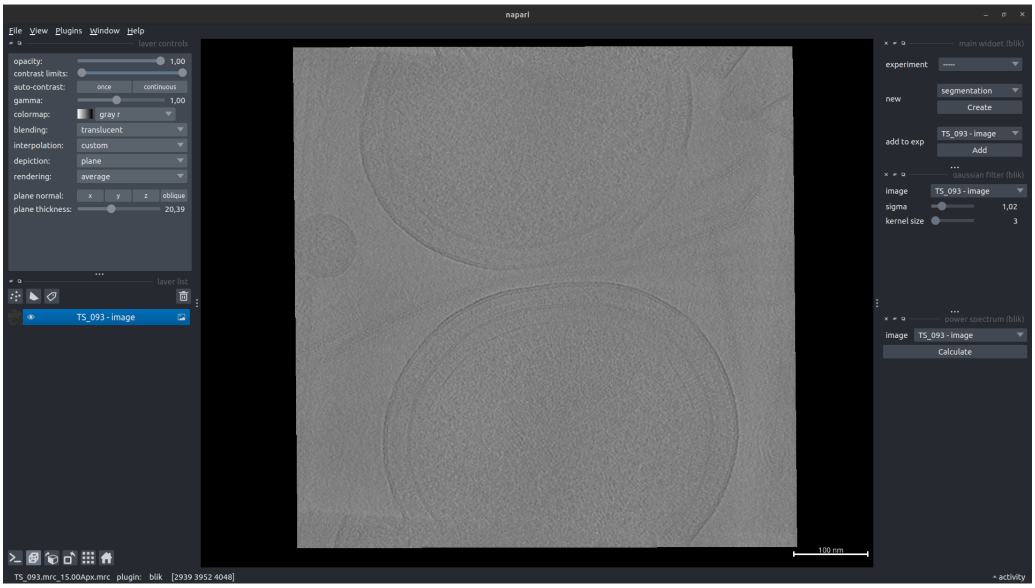


***Figure 5:*** *z_slice through the same tomogram in 3D view with enhanced contrast thanks to averaging over a thick slice.*

with slices oriented along different axes, we can visualise slicing through the tomogram in multiple directions simultaneously (Figure 6).

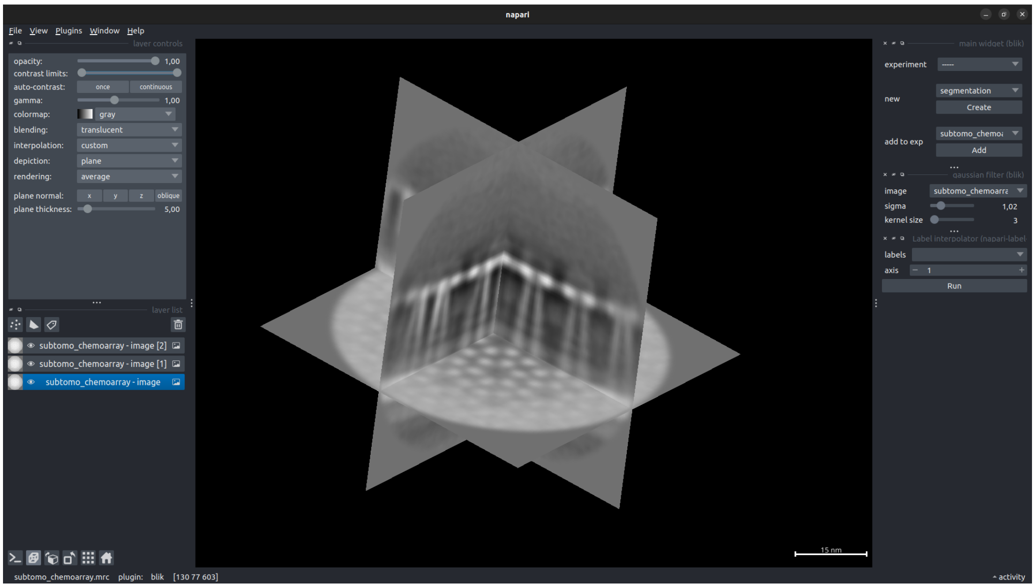


***Figure 6:*** *Three 3D slices along different axes through a map from subtomogram averaging of the chemosensory array.*

To view the full volume in 3D, change the depiction from `plane` to `volume` in the layer controls. A variety of rendering options are available, including isosurfaces (Figure 7) and maximum intensity projections (under `rendering` in the `layer controls`). These options are not exclusive to `blik` but are built-in functions of the `napari` `Image` layer.

`blik` also comes with a widget for quickly visualizing the power spectra of images, slices and volumes (*Plugins > blik > power spectrum*, Figure 8). The power spectrum widget comes with a tick box which lets us input the dimensionality of the data, to distinguish between 3D volumes and stacks of 2D images.

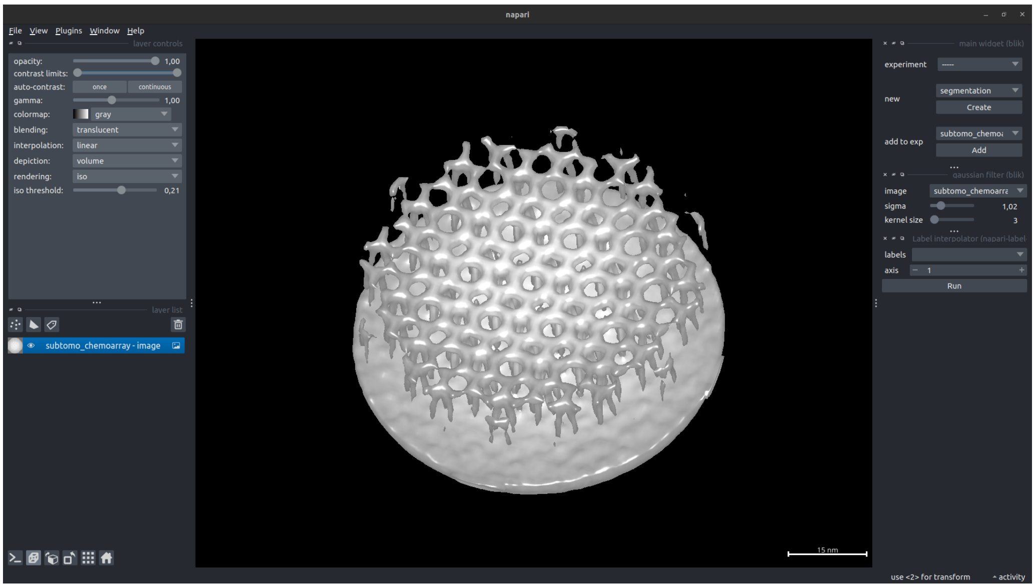


***Figure 7:*** *Visualisation as an isosurface of a map from subtomogram averaging of the chemosensory array.*

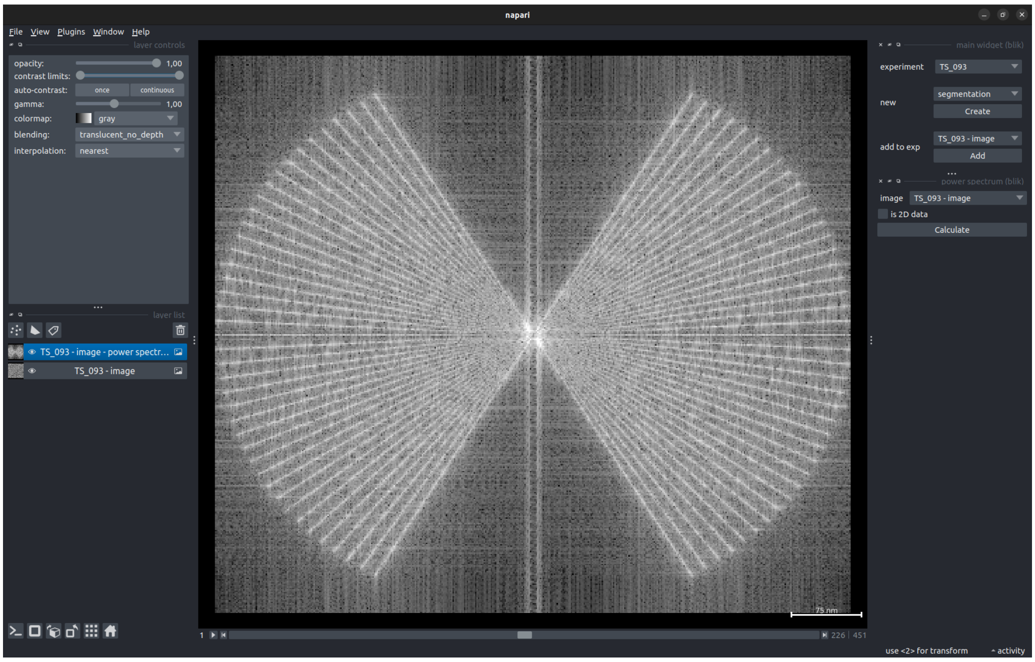


***Figure 8:*** *Z-slice slice through the 3D power spectrum of the tomogram. The central slices from each tilt projection are clearly visible, highlighting the missing wedge characteristic of cryo-ET data.*

### 3.3 Visualising particles

Each particle dataset will be loaded into two separate layers by `blik`: particle locations are loaded as a `points layer`, while orientations will be loaded as a `vectors layer` and by default will be displayed as coloured arrows (Figure 9).

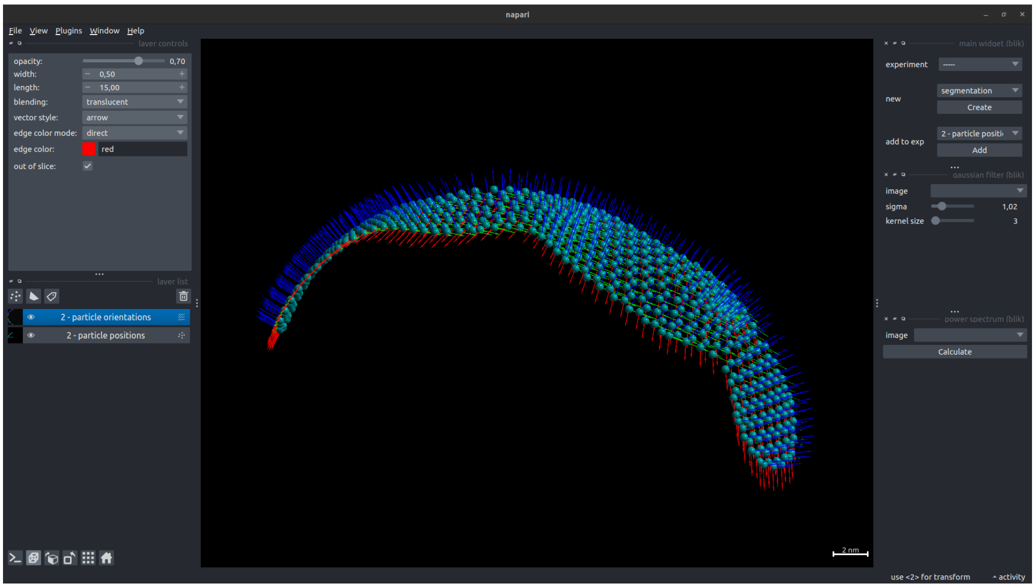


***Figure 9:*** *Positions and orientations of particles picked using vector picking in Dynamo from a surface model. Data loaded from a `.tbl` file.*

Particles can be manually selected by clicking `Select points` from the top of the layer controls panel and then drawing a box around the desired particle. Particles may also be manually created by selecting the `add` tool from the layer controls. The `remove` tool will remove currently selected particles. The appearance of the selected particles may also be changed by using the layer controls. Selecting the orientations layer from the layer list will also let us similarly change the appearance of the orientation arrows.

# 4 Using `blik` to segment and pick

## 4.1 Tomogram segmentation

One of the most common next steps after the generation of a tomogram is the segmentation of the features visible in the data. `blik` comes with its own implemented segmentation function. It can be found under the main widget under `new`. Choose the data which you want to use for segmentation under `experiment` and then create a new segmentation. This will open a labels layers. Here you can adjust parameters for visualization of the segmentation like colour and opacity. By clicking on + and - under `label` you can change between different labels which will all be saved in the same layer

At the top of the layer controls panel, different tools for manual annotation are provided, including the brush for drawing on an image, an eraser and a fill function. Features can be annotated in 2 or 3 dimension, which can be changed using the `n edit dim` function. 3D annotation is currently only available for extending already existing annotations, but full 3D segmentation will be implemented in the future. To interpolate the created labels, we can use the `napari-label-interpolater` plugin. Below you can see a rough segmentation of the cytoplasm of the two cells in our tomogram (Figure 10). The segmentation was created by annotating multiple 2D slices and then interpolating the result.

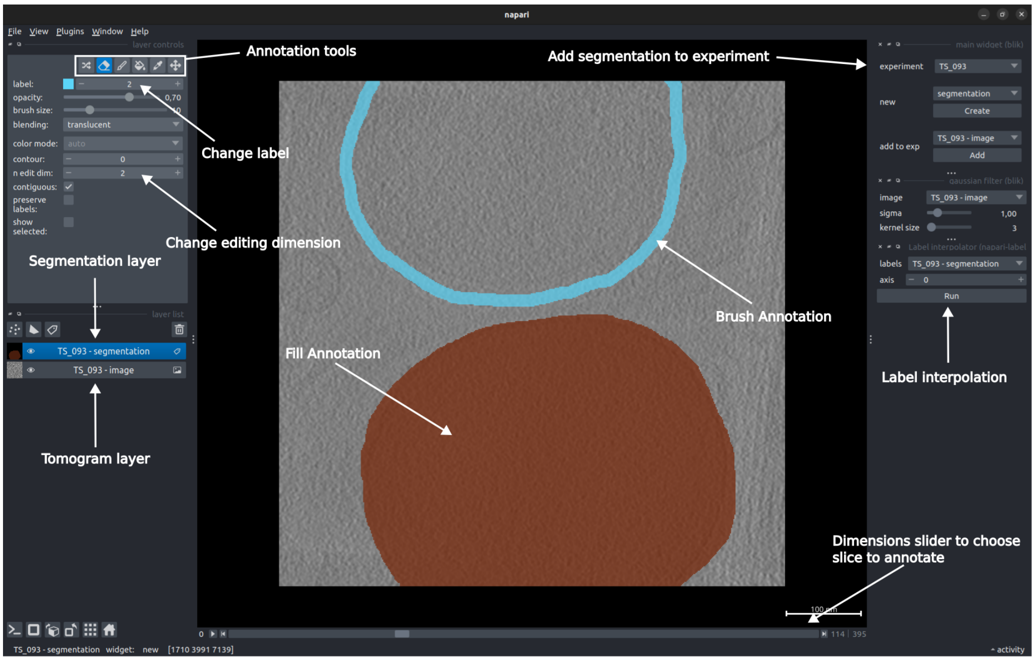


***Figure 10:*** *Rough segmentation of the cytoplasm of the cells in the tomogram. Important parts of the UI are highlighted with arrows.*

To be able to save them with `blik`, interpolated labels must first be added to the experiment to be properly equipped with metadata such as pixel size. We can do so using `add to exp` using the main blik widget widget.

## 4.2 Manual picking

Using the main `blik` widget, we can also create new empty particles for manual picking. This will open two new layers, one for particle positions and one for particle orientations. We can now use the `layer controls` of the `napari Points` layer to add, delete and select particles. By using `add` and clicking on a position in 2D view, we can pick a location, where a new particle will be created. For now, interactive determination of the particle orientation while picking is not possible, so the initial orientation will correspond to all-zero Euler angles.

## 4.3 Surface picking

`blik` also provides tools to pick particles on a surface. In order to define a surface , we can use the `surface picking` option in the main widget. This will create a new `Shapes` layer with many controls; `blik`'s surface generation expects paths created using the `Add path` tool (last button on the top left); using other shapes is currently unsupported.

Using the path tool, we annotate our desired feature in a few 2D slices by left clicking to add points to the `surface lines` layer. The points don't need to be particularly dense: interpolation and smoothing will be applied when generating the surface. By double clicking, each path can be finalised. Repeating this over a few z-slices (about 4 or 5 is usually sufficient for typical surfaces) will define the rough shape of the surface we will generate. If we want to edit one of our points we can select the path on the corresponding z-slice by clicking `select vertices` in the layer controls, and then drag the point to a new position.

Now that we roughly defined the shape of the surface we want to use for picking, we can continue with the surface generation. Open the `surface widget`. By giving our surface lines layer as input to `surface shapes`, we can construct a surface which follows the lines we defined.

The `spacing A` parameter is used to define the spacing (in Angstroms) of individual samples on the surface (Figure 11).

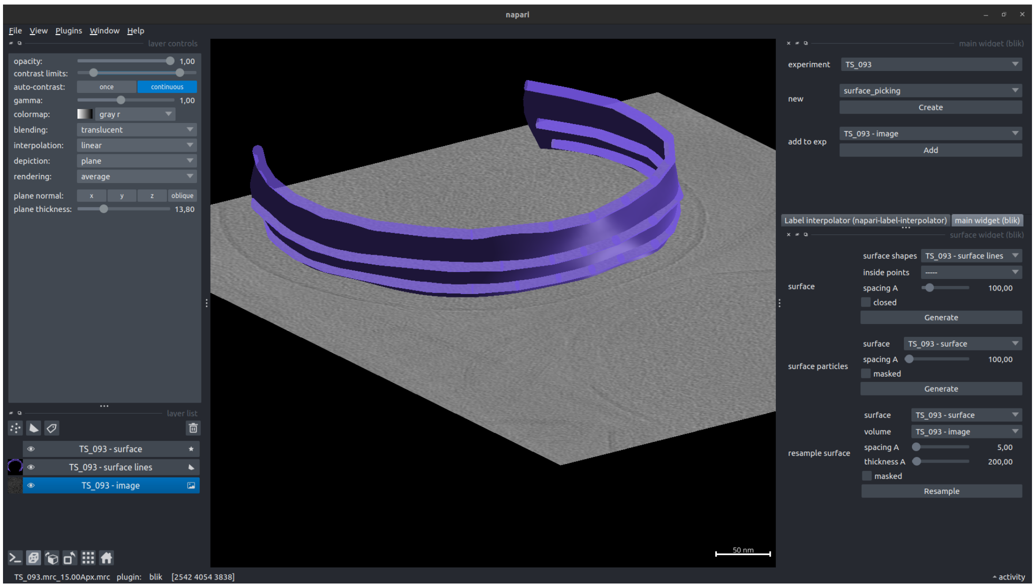


***Figure 11:*** *Surface created from user-defined paths along the inner membrane of the* E. coli *minicells.*

Once we are happy with the shape of the generated surface, we can move on to generating the particles (Figure 12). This is done by using the `surface particle` section. The `spacing A` slider controls the distance between the final particles just as the one above. The orientation of the particles will initialize with the Z axis of the particle orthogonal to the picked surface.

Note that the surface creation algorithm of `blik` can lead to deformed surfaces beyond the edges of the annotation, which may result in unexpected particle orientations; to avoid these artifacts, we can tick `mask` to only create particles strictly within the region of the surface where we defined our paths.

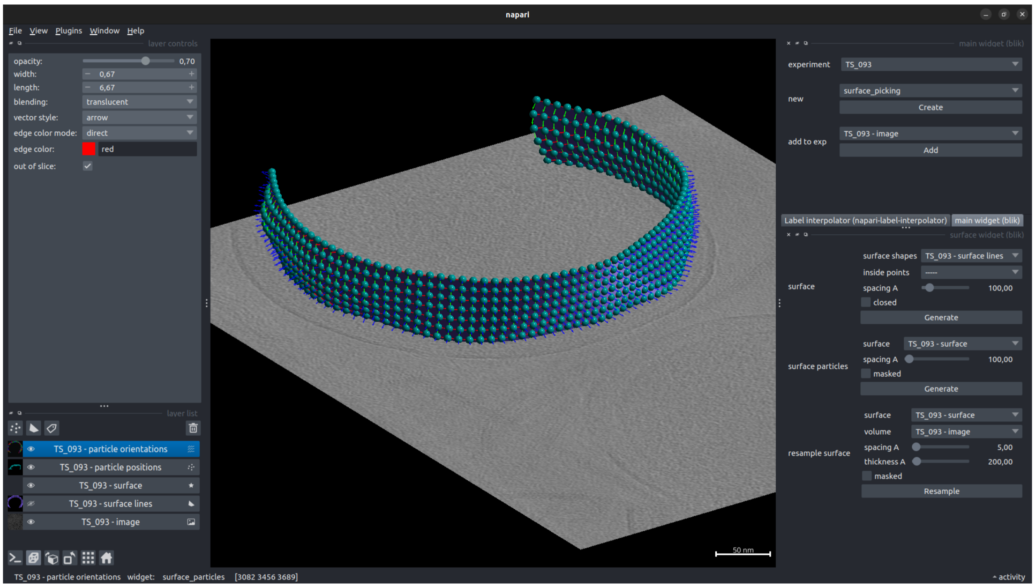


***Figure 12:*** *Particles uniformly distributed on the generated surface.*

## 4.4 Filament picking

Another geometrical picking model implemented in `blik` is the `filament picker`. The procedure is similar to that for surfaces. To start defining a new filament, we need to create a new `filament picking` in the main widget. This will open a new `Points` layer called `filament picks`.

We can now pick coordinates using the `Add points` tool under layer controls. Points can be picked across multiple z-slices (picking in 3D mode is not implemented yet). When we are satisfied with the points, we need to open the `filament widget` to generate the filament from the points which will create a new layer called `filament` (Figure 13).

After the filament itself is created, we need to define the parameters for generating particles along the filament, which are `rise A`, `twist deg` and `radius A`. Information about cyclic symmetry and a twist offset can also be added. The rise parameter will determine the separation distance between the generated particles. Clicking Generate will add new layers which encode particle positions and orientations.

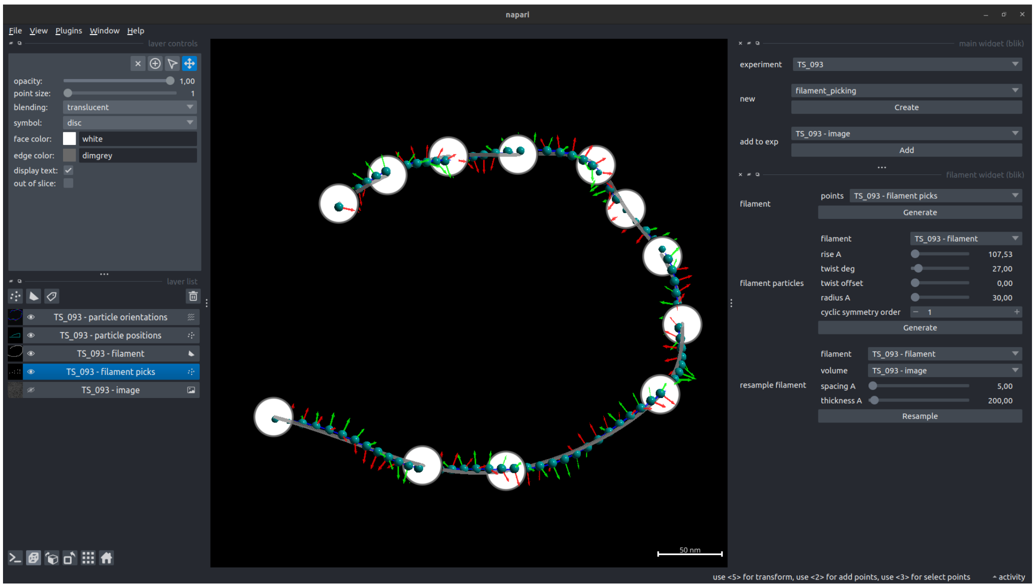


*__Figure 13:__ Particle positions and orientations generated along a helical filament defined by manually picked locations (in white).*

## 4.5 Resampling features

We can also resample a surface using `resample surface` taking a user defined thickness for the resampled volume (Figure 14). This feature comes in handy when we would like to create a spatially consistent volume of a complex 3D object for quantitative analysis. The same as for the surface generation, filaments can also be resampled using the `resample filament` function.

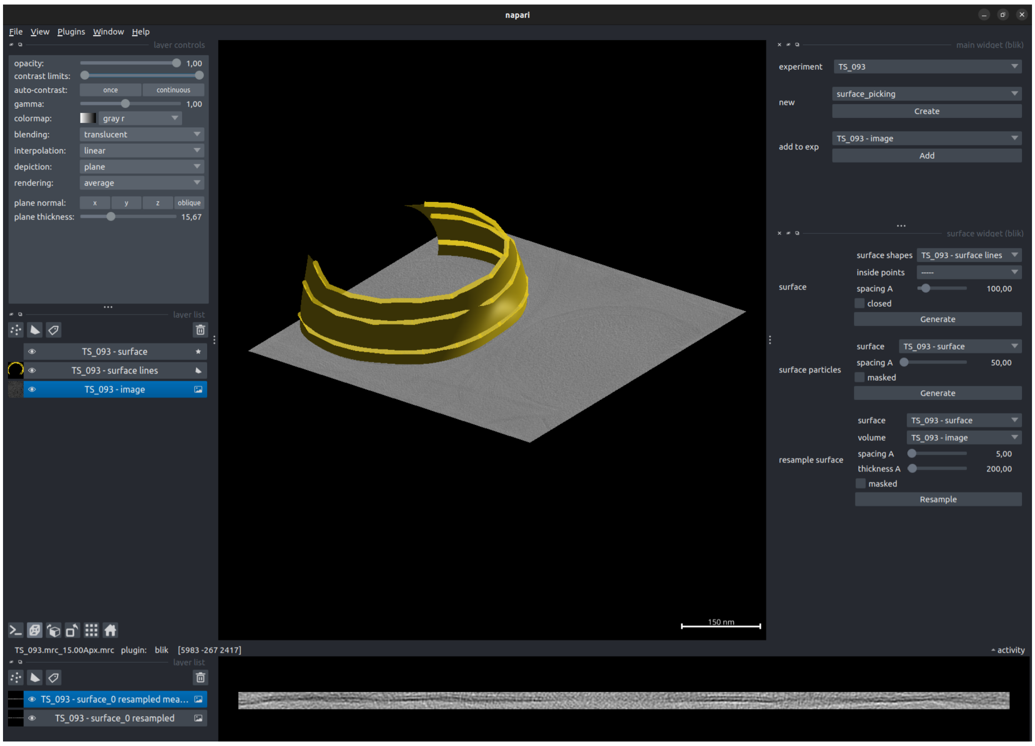


***Figure 14:*** *Resampling of a surface created with the surface picking procedure. The surface was annotated along the outer membrane of an* E. coli *minicell (top), resampled with a thickness of 650 A and then averaged across z (bottom).*

# 5 Inspecting particle metadata

If `blik` was installed with the `[all]` option, it will automatically come with the `napari-properties-plotter` and `napari-properties-viewer` plugins. These can be used for inspecting additional features of particle data directly in the `napari` UI.

The properties-viewer can be used to open a table of all `features` of a layer in `blik`. The features are specific properties of objects in a layer, such as which class each particle belongs to if classification results were present in a loaded `.star` file.

The properties can also be plotted using the properties plotter. Any two features of a layer can be plotted against each other in different styles; choosing just one feature will plot a histogram (Figure 15).

Within the properties-plotter we can choose a selection of data points from the plot by clicking `Select Area`. The specified data can then be interacted with through the layer controls. Below you can see particles loaded from a `.star` file which have been picked using the surface picking protocol. They were equipped with a synthetic feature called “conf”, the values of which follow a normal distribution, which has been plotted against the particle index using the properties-plotter. Data points with “conf” higher or equal 1 have been selected from the plotter and were coloured red using the layer controls.

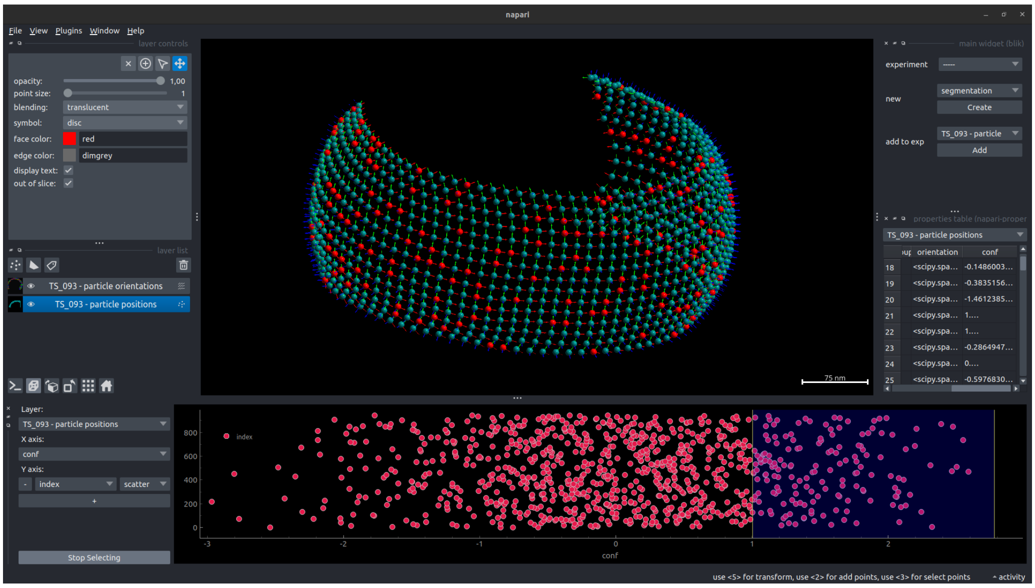


***Figure 15:*** *Using the properties plotter to select and interact with particles with the desired feature values.*

# 6 Creating custom widgets

`napari` integrates with a tool called `magicgui` to make it easy to create simple custom widgets to interact with the viewer and the data by writing simple python code. A thorough explanation can be found in the `napari` documentation for magicgui. Here's a (complete!) example showcasing how to create a slider to control the threshold of a simple segmentation algorithm, which allows to quickly and interactively find the best value for the parameter.

```python
# segment_with_napari.py

import napari
from magicgui import magicgui

from skimage import data
from skimage.filters import threshold_otsu
from skimage.measure import label
from skimage.morphology import closing, remove_small_objects, square
from skimage.segmentation import clear_border

# call the function automatically on any change and use a slider widget
@magicgui(
    auto_call=True,
    threshold=dict(widget_type='FloatSlider', min=0, max=300)
)
def segment(
    image: napari.layers.Image,  # autodetect image layers as inputs
    threshold: float = 50,
) -> napari.types.LayerDataTuple:  # autoadd the output to the layerlist
    # see scikit-image docs for details on these functions
    thresholded = closing(image.data > threshold, square(4))
    cleared = remove_small_objects(clear_border(thresholded), 100)
    label_image = label(cleared)

    return label_image, {'name': 'result'}

# load sample data and open it in napari
image = data.coins()[50:-50, 50:-50]
viewer = napari.view_image(image, name='coins', rgb=False)

# add the widget to the viewer window
viewer.window.add_dock_widget(segment)

napari.run()
```
