# Supplementary material for: Blik is an extensible 3D visualisation tool for the annotation and analysis of cryo-electron tomography data
Source: PLoS Biol. 2024 Apr 30;22(4):e3002447. doi: 10.1371/journal.pbio.3002447 (PMC11268629; doi:10.1371/journal.pbio.3002447)
Supplement: Attachment — Submitted filename: point-by-point-240307-final.pdf [file pbio.3002447.s002.pdf]

Point-by-point response.

Reviewer #1: In this manuscript, Gaifas et al describe a new Napari-based tool for visualisation, annotation and analysis of cryo-ET data, which they name blik.
Their software aims to be compatible with commonly used cryo-ET processing packages and provide a comprehensive suite of useful tools. As such, blik should significantly lower the energy required for projects where switching between different software is necessary, and would be a very welcome addition to the fast-growing cryo-ET field.

We thank the reviewer for their recognition of the value provided by our work.

The paper is clearly written, however, some clarifications are needed. Some additional capabilities and tutorials should also be included before publication. Details of required revisions below.

Page 2, line 18-19. emclarity should be cited, as well as pytom/the av3/tom toolbox. While some of these are becoming obsolete, many of the tools currently used are still based on file formats from these software. Also the authors cite imod but also peet should be included.

Citations for these software suites were added.

Page 2, line 53 (and further references later). The authors refer to relion, but they need to clarify which relion. The current warp/M pipeline uses relion 3, while a self-sufficient modern subtomogram pipeline is offered in relion 4 and 5, however these are not compatible in terms of file format, and blik needs to be able to read and write star file compatible with relion 3 (to maintain compatibility with Warp/M) as well as 4/5.

Specific Relion versions were not referred to because they are all supported seamlessly. This point is now specifically mentioned in the manuscript in the Input/Output section.

Page 4, line 108. Fig 1E shows a slice through a power spectrum, not a gaussian filtered image as described.

We thank the reviewer for pointing this out, we realise that the text was out of sync with the image update. It is now updated to reflect the correct figure.

Figure 2 and 'Particles' section. The depiction is beautiful and clear. However, there are tools out there such as 'place objects in space' from the Briggs lab which are widely used (and are based on the tom/av3 motivelist format), which currently offer to plot not just spheres and arrows, but also other geometrical shapes such as square, rectangles, triangles, hexagons etc. This is incredibly useful and should be added to blik.

In fact, blik has these features as well because showing points as different symbols (such as squares, stars, crosses, etc) is already implemented as a builtin feature in napari. Improvements to this rendering were actually part of the work for blik, but this was not mentioned in the paper because the changes done by us were minor (allowing per-point, feature-based symbols rather than just one symbol for all points in a layer).

Moreover, it is possible to plot any map (such as the subtomogram average). I appreciate this is described as part of the future developments in the discussion, however the authors only cite the recently released ArtiaX as their inspirations, while the tool has been freely available from the Briggs lab since 2018.

Indeed, we are aware that the Briggs lab created a variety of extremely useful tools. To our knowledge, many of them are used mostly in-house or by direct collaborators. Thus, we were not aware of this particular tool and were indeed inspired by ArtiaX, as we acknowledge in the manuscript.

Filaments section. This should include citations to the works from Briggs which also include filament pickers working in a very similar manner. (and which are also provided for spherical objects). Both here and in the surface section, more explicit reference to dynamo should be made. I appreciate a general statement of 'inspiration' is present in the discussion, but I think the authors should specifically mention which tools their ideas are based on.

Thank you for the suggestion. Since we are actively using Dynamo in our research, it does indeed significantly imprint on the the trajectory of our own developments. As much as we enjoy working in Dynamo, it is MATLAB-based, and we think that Python, with its open-source nature, wide adoption by young researchers, and ease of development, makes it a better choice for scientific tooling. This was one of the main reasons for the development of blik in the first place. We now make the influence of Dynamo more explicit in the beginning of the filament and surface generation section as requested. Yet, we must admit that the tools by the Briggs group that you mention were unknown to us at the time of writing, and to date we cannot find any clear reference, documentation, or source code in the literature regarding such tools. We will be happy to get access to these tools which will certainly serve as an inspiration for our further research and development. As for the present manuscript, all software that stimulated initial development and evolution of blik have been acknowledged.

Resampling: again a very nice tool, which is great to have within the same suite as all the others, but the section lacks reference to previous work from Dimitry Tegunov (membranorama).

Membranorama by Dimitry Tegunov is a very different tool. Its focus is visualisation, as no output volume is generated for further processing and data is never accessible to the user. Even when visualising surface patches in Membranorama, the way “planarization” works is very different: no attempt is made to maintain a quasi-square grid which is essential to obtain a sensible volume array for further processing. A short reference to membranorama and an explanation of these differences is added at the end of the Discussion section.

Lastly, but most importantly, I have installed blik and had a quick play with it, and I find that it is not intuitive at all, especially for users who are not familiar with Napari (which is most people). For example, I was not able to work out how to display isosurfaces (which I should hope is possible). A step-by-step tutorial is required.

We are very grateful to you for this excellent suggestion. We have now written a detailed tutorial which explains the basics of napari, also pointing to the full documentation where relevant. It also explains how to use each main feature of blik for practical purposes with some examples. This tutorial is provided as a supplementary file and is also now accessible from the blik documentation website, where it will be kept up-to-date with the software. We felt that the best way to satisfy all concerns regarding the user-friendliness of blik and to assure the broad accessibility of the tutorial, was to ask our colleague Moritz Kirchner, a biochemist with no prior expertise in napari and a beginner in cryo-electron tomography, to use blik on a test data set, discover its most important functionalities and potential pitfalls, and carefully describe each of the steps. We are very pleased with the outcome, not only because this exercise resulted in a very comprehensive tutorial, but also because it helped identify a number of small bugs and usability issues that are now corrected, thus improving the quality of our plugin. Considering the extensive effort provided by Moritz Kirchner in preparing and thoroughly testing the tutorial and providing feedback, we include him in the author list to acknowledge his contribution to this publication.

Reviewer #2: Summary:
This manuscript describes the features of blik, an open-source Napari plugin for the visualization and annotation of cryo-electron tomography (cryo-ET) data. The authors demonstrate how blik builds on available functionality in Napari for cryo-ET data visualization by adding new features, such as 3D volume filters (e.g., gaussian filter, 3D power spectrum), particle data (e.g., location, orientation), image segmentation (e.g., volumetric voxel labeling), filament tracing, and surface generation. The authors demonstrate how output/input files from disparate cryo-ET software (e.g., dynamo, relion, imod) can be easily read and opened in blik. The authors showcase filament- and

surface-based particle picking and resampling, akin to other methods, but distinguishably advantageous in blik due to its Python-based scripting.

We thank the reviewer for their positive evaluation of our work and for recognition of the advantages of using the scientific Python ecosystem.

Significance:
This manuscript tackles a crucial requirement in the cryo-ET field: the development of software that seamlessly integrates the visualization of output data from diverse sources into a unified platform. Due to the 3D nature of tomographic data, it is often difficult to visualize complex spatial relationships between different subcellular structures simultaneously. Napari visualization software laid the groundwork for handling cryo-ET data, and blik expands upon this foundation by integrating additional data analysis outputs commonly associated with such studies (e.g., segmentations, particle information, etc.). While there are still additional functionalities that the tomography field will likely want to incorporate, this software represents precisely the kind of tool the field desperately needs. I believe it marks an essential first step in the right direction.

We are very thankful for your analysis and will definitely continue development of blik and incorporation of additional functionalities. We hope that publication of this work should increase the user community of blik, who will come up with their feedback and suggestions for further improvement.

Overall, this manuscript does a good job of describing the various functionality of blik with specific examples of the implementation of its different features visualized in the corresponding figures. Below are a few suggestions to improve the overall clarity and flow of the manuscript:

1. All the figure legends could benefit from more clarity. For example, figure 5 cannot stand alone with the current legend without referencing the relevant main text portion. The manuscript would also benefit from additional references to specific panels of the figures in the main text. Additionally, some of the figure panel references seem to be in the wrong place; for example, a reference to Figure 1E in line 108 may be better suited for the later section in line 119.

We fully agree, we clearly did not pay enough attention to the self-sufficiency of the figure captions in the first version of the manuscript. We now expanded the figure legends and made clear references to different figure panels in the text. The reference to figure 1E was mistakenly left over from a previous version of the image, and is now in the correct section.

2. The properties plotter plugin is super useful and exciting; however, the example given in Figure 2 may not be the best representation of the powerful utility of this tool. Index versus rlnLogLikeliContribution value seems like a poor representation that does not have much intuitive value. Perhaps it would be better to show a histogram of the values that give clear selection or scoring criteria, much like what is implemented in other software such as Pytom or Artiax. The authors mention in a later section, starting in line 267, "Particles may hold extra metadata such as classification results or quantitative values such as confidence score." It would be better to show a parameter like this in Figure 2.

Thank you for the suggestion, we have now updated the Figure 2 and show classification results plotted as a histogram, which indeed better showcases the utility of the tool. The new caption mentions some insights that can be gained through its use. The main text was updated to clarify that the plotter can be used also to generate X-Y scatter plots with combinations of features.

3. Related to the previous point, Lines 267-273 seem better suited in the earlier Particles section.

These lines were removed and partially integrated in the earlier Particles section as suggested.

4. The discussion is relatively thorough in comparing Blik to other currently available cryo-ET software for simultaneous visualization of tomograms, segmentations, particle picks, etc. However, I believe it is worth mentioning other available software for generating and visualizing filament and membrane segmentations (i.e., tomomemsegtv, membrain-seg, dragonfly), density resampling (i.e., membranorama), and surface mesh reconstructions (i.e., pycurv, surface morphometrics).

We slightly expanded the discussion and hope that this satisfies the reviewer concern to some extent. We prefer however not to try (and fail) to make a list of all existing software and to stay deliberately non-exhaustive, as new software becomes available every day, and this paper is not intended to be a review of the field.
